# Recombinant *O*-mannosylated protein production (PstS-1) from *Mycobacterium tuberculosis* in *Pichia pastoris* (*Komagataella phaffii*) as a tool to study tuberculosis infection

**DOI:** 10.1186/s12934-019-1059-3

**Published:** 2019-01-19

**Authors:** Giroshi Bando-Campos, Daniel Juárez-López, Sergio A. Román-González, Antonia I. Castillo-Rodal, Clarita Olvera, Yolanda López-Vidal, Roberto Arreguín-Espinosa, Clara Espitia, Mauricio A. Trujillo-Roldán, Norma A. Valdez-Cruz

**Affiliations:** 1 Programa de Investigación de Producción de Biomoléculas, Departamento de Biología Molecular y Biotecnología, Instituto de Investigaciones Biomédicas, Universidad Nacional Autónoma de México, AP. 70228, CP. 04510 Ciudad de México, Mexico; 2 Unidad de Proteómica, Instituto Nacional de Medicina Genómica (INMEGEN), Periférico Sur 4809, Col. Arenal Tepepan, Tlalpan, C.P. 14610 Ciudad de México, Mexico; 3 Programa de Inmunología Molecular Microbiana, Departamento de Microbiología y Parasitología, Facultad de Medicina, Universidad Nacional Autónoma de México (UNAM), 04510 Ciudad de México, Mexico; 4 Departamento de Ingeniería Celular y Biocatálisis, Instituto de Biotecnología UNAM, Av. Universidad 2001 Chamilpa, Cuernavaca, Morelos Mexico; 5 Departamento de Química de Biomacromoléculas, Instituto de Química, Universidad Nacional Autónoma de México, Av. Universidad 3000, Ciudad Universitaria, Apdo, Postal 70250, C.P. 04510 México City, Mexico; 6 Departamento de Inmunología, Instituto de Investigaciones Biomédicas, Universidad Nacional Autónoma de México, Ciudad de México, Mexico; 7 Programa de Investigación de Producción de Biomoléculas, Unidad de Bioprocesos, Departamento de Biología Molecular y Biotecnología, Instituto de Investigaciones Biomédicas, Universidad Nacional Autónoma de México, AP. 70228, CP. 04510 Ciudad de México, Mexico

**Keywords:** *Mycobacterium tuberculosis*, Antigen, Glycoprotein, *Pichia pastoris*, PstS-1, *O*-mannosylation, *Komagataella phaffii*

## Abstract

**Background:**

*Pichia pastoris* (syn. *Komagataella phaffii*) is one of the most highly utilized eukaryotic expression systems for the production of heterologous glycoproteins, being able to perform both *N*- and *O*-mannosylation. In this study, we present the expression in *P. pastoris* of an *O*-mannosylated recombinant version of the 38 kDa glycolipoprotein PstS-1 from *Mycobacterium tuberculosis* (*Mtb*), that is similar in primary structure to the native secreted protein.

**Results:**

The recombinant PstS-1 (rPstS-1) was produced without the native lipidation signal. Glycoprotein expression was under the control of the methanol-inducible promoter pAOX1, with secretion being directed by the α-mating factor secretion signal. Production of rPstS-1 was carried out in baffled shake flasks (BSFs) and controlled bioreactors. A production up to ~ 46 mg/L of the recombinant protein was achieved in both the BSFs and the bioreactors. The recombinant protein was recovered from the supernatant and purified in three steps, achieving a preparation with 98% electrophoretic purity. The primary and secondary structures of the recombinant protein were characterized, as well as its *O*-mannosylation pattern. Furthermore, a cross-reactivity analysis using serum antibodies from patients with active tuberculosis demonstrated recognition of the recombinant glycoprotein, indirectly indicating the similarity between the recombinant PstS-1 and the native protein from *Mtb*.

**Conclusions:**

rPstS-1 (98.9% sequence identity, *O*-mannosylated, and without tags) was produced and secreted by *P. pastoris*, demonstrating that this yeast is a useful cell factory that could also be used to produce other glycosylated *Mtb* antigens. The rPstS-1 could be used as a tool for studying the role of this molecule during *Mtb* infection, and to develop and improve vaccines or kits based on the recombinant protein for serodiagnosis.

**Electronic supplementary material:**

The online version of this article (10.1186/s12934-019-1059-3) contains supplementary material, which is available to authorized users.

## Background

Pulmonary tuberculosis (TB) is caused by the bacterium *Mycobacterium tuberculosis* (*Mtb*) and is the second most prevalent infectious disease in the world. In 2016, the World Health Organization reported 10.4 million new TB infections, and 1.3 million deaths due to disease complications, in addition to the fact that about a quarter of the world population is suspected to be infected with the mycobacterium in a clinically latent form [1]. In the same year, it was estimated that approximately 600,000 people developed multidrug-resistance (MDR) to TB [1]. These data reflect the urgency in understanding the pathogenicity of *Mtb*, as well as the necessity to develop new therapeutic alternatives to treat or prevent the disease. The interaction of this bacillus with the host has been extensively studied for a long time, with the research effort focusing on its virulence factors, such as antigens [2–8]. The production of recombinant *Mtb* antigens is important in several aspects of Mtb research, for example, in studying antigen interactions and their effect in vivo and in vitro [9–13], in the designing effective vaccines against *Mtb* [14, 15], as well as in developing diagnostic tools for TB [2, 3, 8, 16, 17]. In fact, the use of recombinant antigens to detect both latent and active TB provides for a simpler and more accurate diagnosis compared to other diagnostic tests in use today, such as the purified protein derivative (PPD) skin test or the QuantiFERON^®^-TB test [18–21]. For these reasons, it is important to be able to produce antigens with conformations that are as similar as possible to the native antigens produced by the mycobacteria so they can used as reagents in the development of diagnostic tools or vaccines.

*Mtb* produces a variety of secreted protein antigens and these can be found in culture supernatants. Some of these, such as Rv2164c, Rv3491, Rv0175, Rv1887, Rv1096, Rv2068c, Rv2744c, Rv2799, Rv3835, and Rv1860, among others, are modified by the addition of mannose residues [22–25]. This post-translational modification could contribute to virulence, colonization, and invasion of the host cell [16, 22, 26, 27]. However, the significance of the *O*-mannosylation of glycoproteins in infection and the host innate immune response is poorly understood. Previously, it has been demonstrated that the *O*-mannosylation pattern of the alanine and proline-rich protein (APA), which is a immunodominant secreted antigen from *Mtb*, determines its activity [24, 28, 29]. Native APA induces a potent, delayed-type hypersensitivity response, and stimulates the priming of T-cells in vitro and in vivo, in contrast to the non-glycosylated recombinant APA produced in *E. coli* [28, 29]. Moreover, antibodies derived from human TB patients react strongly with the *O*-glycosylated form of APA, whereas the non-glycosylated protein is unable to bind to these antibodies [30]. In addition, it has been suggested that the protective properties of the anti-tuberculosis vaccine are related to the pattern of *O*-mannosylation generated by *M. bovis* bacillus Calmette-Guérin [27–29, 31]. Other *O*-mannosylated proteins are also potent *Mtb* antigens such as PstS-1, LpqH, and LprG [23, 32, 33], in which their glycan structures have been suggested to interact with host receptors, such as DC-SIGN on human dendritic cells [34], Toll-like receptors [35], and mannose receptors [36].

The *Mtb* phosphate-binding protein PstS-1 (Rv0934, PhoS1 or PBP-1) is an antigenic glycolipoprotein that is produced and secreted by *Mtb*. This 38 kDa antigen is composed of 374 amino acids including a signal peptide that is proteolytically removed to generate a mature protein of 351 residues, which is then exported to the outer membrane surface of *Mtb* [37–39]. This antigen belongs to the ABC type phosphate transport system [40, 41] and its accumulation in the cell wall increases in response to an absence of phosphate in the culture medium [41]. Importantly, this protein induces a strong immune response and causes adaptive protective immunity in mice [42, 43] and humans [38, 44]; furthermore, it has been reported to be associated with the active form of TB [45, 46]. Although it is known that the native antigen is also *O*-mannosylated [23, 36], the exact sites of its *O*-glycosylation remain unclear. Using a predictive tool to analyze several *Mtb* glycoproteins, it has been proposed that PstS-1 has three *O*-glycosylation sites, most probably at threonines 20, 21, and 28 at the N-terminus [47]. To date, the crystallographic structure of native PstS-1 has not been determined.

In yeasts such as *S. cerevisiae, O*-mannosylation is also an important post-translational protein modification, playing roles in the composition of cell walls, cellular differentiation, septation and viability in fission yeast, vesicle delivery, and virulence [48–51]. *O*-linked mannosylation in yeasts occurs through the attachment of mannose glycans to a serine or threonine residue on substrate proteins entering the ER-Golgi pathway, through a series of mannosyltransferases [52–54]. Interestingly, the *O*-mannosylation pathways between yeast organisms and actinomycetes like *M. tuberculosis* are evolutionarily conserved [53]. *O*-mannosylation in actinomycetes, occurs in a manner such that mannose residues are α-1,2-linked to serine or threonine residues on proteins residing in bacterial membranes [55–58]. Similarly, in *Pichia pastoris,* the *O*-linked glycans produced are linear chains of four to five α-1,2-linked mannose residues [56, 59], whereas *S. cerevisiae* generates α-1,2 and α-1,3-linked mannose residues [60–62] in the highly *O*-mannosylated form [53]. Hence, the methylotrophic yeast *P. pastoris* is preferred over *S. cerevisiae* as a heterologous system to produce glycoantigens from *Mtb*, because their mannose chains are shorter [63], and mimics the modifications performed by *Mtb*. This was demonstrated during the production of the antigen rCFP32 in *P. pastoris*, which, compared with the rCFP32 produced in *E. coli,* resulting in a rCFP32 protein that was majorly immunoreactive, as assessed by in vitro antibody production and the serum titers from tuberculosis patients [64, 65]. Furthermore, the use of a heterologous system based on the methylotrophic *P. pastoris* results in a high productivity for recombinant proteins under conditions that are free from endotoxins and viral DNA [66–68]. *P. pastoris* is thereby now considered a safe organism, in which several human biopharmaceuticals have been produced [69].

Thus, with the intention of producing the glycoantigen PstS-1 with post-translational characteristics similar to that of the native antigen produced by *Mtb*, and to avoid the use of harmful *Mtb* which requires long cultivation periods, we describe here the production of a non-tagged recombinant *O*-mannosylated glycoantigen PstS-1. This work describes the production on the laboratory scale (shake flasks and bioreactors) of a recombinant form of the *O*-mannosylated glycoantigen PstS-1 (rPstS-1, 98.9% sequence identity to the native protein), its purification and characterization, some of its *O*-mannosylation modifications, and its immunological reactivity by sera from patients diagnosed with tuberculosis. The production of this glycoprotein will aid in the study of its immunological activity and will be useful as a diagnostic tool and/or as a vaccine against TB.

## Results and discussion

### Development of strains secreting rPstS-1

The gene sequence encoding *M. tuberculosis* PstS-1 (Rv0934) (GenBank number: P9WGU1) was synthesized, accommodating for the preferential codon usage in *P. pastoris* [70, 71]. The synthesized DNA also excluded the nucleotide sequences encoding amino acids 1–21 (MKIRLHTLLAVLTAAPLLLAA) of the 23 amino acids that form the signal peptide. The two amino acids in the signal peptide that were retained were Ala-Gly (22-23). The retention of these avoids lipidation of the Cys residue (residue 24), which would normally be present at the N-terminus of the mature processed protein. Moreover, since two amino acids (N57 and N247), were predicted to be potential N-glycosylation sites (NetNGlyc 1.0.), these residues were substituted with glutamines (N55Q and N247Q) to maintain a conformation similar to that of native PstS-1 [72, 73]. The synthesized coding sequence was then cloned downstream of the α-mating factor in the vector pPICZαB, conserving the open reading frame, and under the control of the *P. pastoris* alcohol oxidase 1 promoter (pAOX1). This promoter is tightly regulated by methanol; however, its transcriptional regulatory mechanism has only partially been described [74]. Induction of the pAOX1 promoter requires the lack of a repressing carbon source, such as glycerol; the depletion of such a carbon source results in a slight de-repression of the promoter. In addition, methanol (the inducible carbon source) causes significant pAOX1 de-repression, normally resulting in production of the enzymes required for methanol utilization [66, 74–76]. Here, rPstS-1 was expressed without tags because these might alter the biological or physicochemical properties compared to native or unlabeled proteins [77–80]. The constructed plasmid was verified by PCR and sequencing. Since *P. pastoris* X-33 was transformed with this recombinant plasmid, *P. pastoris* X-33 was also transformed with the empty plasmid vector as a control. YPD agar medium supplemented with Zeocin™ was used to select for recombinant yeasts. The selected clones were cultured in a 50 mL BMGY culture which was then used to inoculate a 250 mL culture in BSF, and after 32 h in BMMY media and following the addition of 5, 10, or 20 mL/L of methanol, the growth and production of rPstS-1 were compared. After 60 h of culture an increase in production of approximately 15–20% was observed when adding 10 mL/L methanol, compared to 5 and 20 mL/L of methanol (data not shown). As previously reported, the methanol concentration appears to affect the productivity depending on the clone [81].

### Use of baffled shake flasks and bioreactor cultures of P. pastoris to produce rPstS-1

**Fig. 1:**
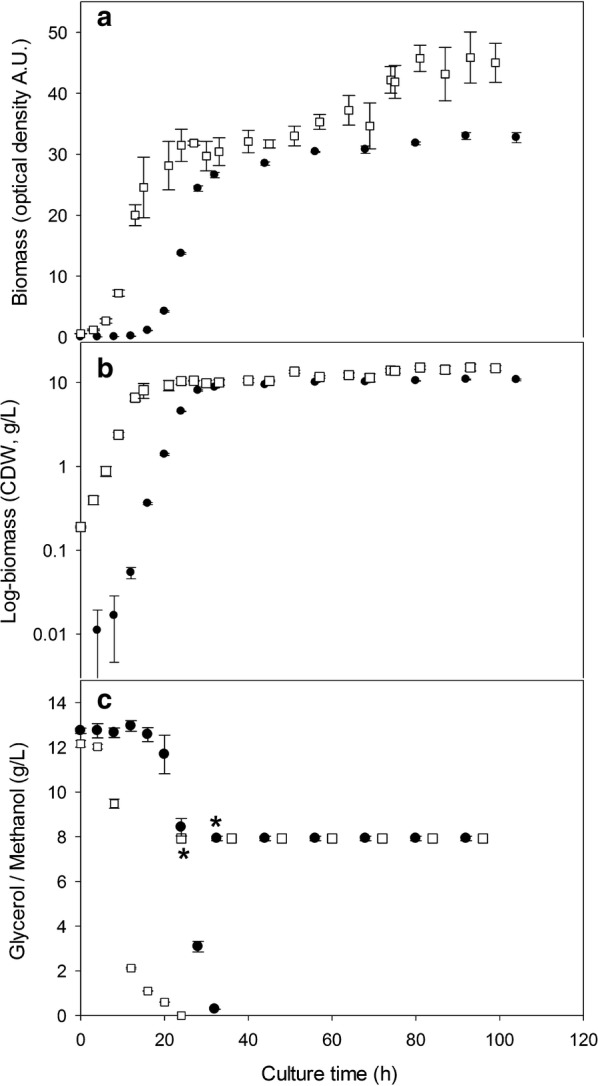
Kinetic growth of the recombinant strain *P. pastoris* producer of rPstS-1 measure as optical density (**a**) and dry weight (**b**) and carbon source consumption (**c**), batch phase glycerol was measured and the feeding by methanol was also shown (start of feeding/induction was marked, upper asterisk for baffled shake flasks, down asterisk for bioreactor), in baffled shake flask (close dots) and controlled bioreactor (open squares)

The *P. pastoris* X-33 clone producing rPstS-1 was characterized with regard to its growth in 50 mL of BMGY in BSFs at 30 °C and 250 rpm. After complete glycerol consumption (32 h), all the cells were transferred to 50 mL of BMMY containing 1% methanol (10 mL/L), which was fed every 12 h, and followed until 73 h post induction. In BMGY media, the maximal biomass reached was 26.6 ± 0.4 A.U. (8.8 ± 0.2 g/L), with a pre-induction specific growth rate of 0.323 ± 0.025/h (Fig. 1a, Table 1). In BMMY, used to induce rPstS-1 expression, the maximal biomass achieved was 32.7 ± 0.8 A.U. (10.8 ± 0.3 g/L) (Fig. 1a, Table 1), with a post-induction specific growth rate of 0.003 ± 0.001/h (Table 1). Approximately a 23% higher level of biomass accumulated after induction. A specific glycerol consumption rate (q_s_) of 0.021 ± 0.004 g_Glyc_/g_DCW_ h and a yield of biomass per glycerol consumed of 0.69 ± 0.01 g_DCW_/g_glycerol_ were obtained. The biomass to methanol yield was 0.051 ± 0.004 g_DCW_/g_methanol_.

**Table 1:** Stoichiometric and kinetic parameters of *P. pastoris* growth and rPstS-1 production cultured in baffled shake flasks and in 1.0 L bioreactors

Parameter	Baffled shake flask	Bioreactor
Max O.D. 600 nm glycerol (A.U.)	26.5 ± 0.4	31.5 ± 2.2^b^
Max O.D. 600 nm methanol (A.U.)	32.7 ± 0.8	47.0 ± 2.6^a^
Max biomass glycerol (g/L)	8.8 ± 0.2 g/L	10.4 ± 0.9^b^
Max biomass methanol (g/L)	10.8 ± 0.3 g/L	15.5 ± 0.9^a^
μ glycerol (/h)	0.323 ± 0.025	0.286 ± 0.033
μ methanol (/h)	0.003 ± 0.001	0.006 ± 0.001^b^
Y_x/s_ (g_DCW_/g_Glyc_)	0.69 ± 0.01	0.81 ± 0.03^b^
Y_x/s_ (g_DCW_/g_Meth_)	0.051 ± 0.004	0.092 ± 0.004^a^
q_s_ (g_Glyc_/g_DCW_ h)	0.021 ± 0.004	0.034 ± 0.013
Supernatant total protein (mg/L)	127 ± 15	185 ± 9^a^
Y_STP/DCW_ (mg _STP_/g _DCW_)	11.75 ± 1.13	11.92 ± 0.59
rPstS-1 (mg/L)	46 ± 5	46 ± 4
q_rPstS-1_ (mg/L h)	0.639 ± 0.070	0.633 ± 0.054
q_p(rPstS-1)_ (mg_rPstS-1_/g_DCW_ h)	0.059 ± 0.002	0.040 ± 0.004^a^
Y_rPstS-1/x_ (mg_rPstS-1_/g_DCW_)	3.3 ± 0.01	4.0 ± 0.02^a^

The mean and standard deviation for at least three biological replicates are shown

*O.D.*, optical density; A.U., absorbance units; µ, specific growth rate; STP, supernatant total protein; DWC, dry cell weight; Y_x/s_, total dry cell weight yield per glycerol; Y_x/s_, total dry cell weight yield per methanol; Y_STP/DCW_, STP yield per DCW; q_s_, specific consumption rate of glycerol per DCW; q_PstS-1_, volumetric productivity of rPstS-1; q_p(rPstS-1)_, specific productivity of rPstS-1; Y_rPstS-1/x_, rPstS-1 yield per DCW. µ was calculated in first 8 h of culture

*p* value was calculated using *t* test by comparing the results of Shake flask VS Bioreactor

^a^*p* < 0.005; ^b^ *p* < 0.05

The *P. pastoris* X-33 clone producing rPstS-1 was also characterized in bioreactor cultures. For this, a typical feeding profile for a carbon source was used; this started with a batch culture in glycerol (BMGY), and when an increase in the dissolved oxygen tension (DOT) was observed (as a result of the exhaustion of the carbon source), the methanol fed-batch phase was started. This strategy was followed to avoid methanol accumulating to high levels, which could cause the accumulation of formaldehyde to toxic levels [82, 83]. Using glycerol as a carbon source, a maximal biomass of 10.4 ± 0.9/gL was obtained (Fig. 1, Table 1). Glycerol depletion was observed after 24 h of culture. After consumption of the glycerol, methanol feeding (10 mL/L) was performed every 12 h to induce expression of the rPstS-1 (Fig. 1c). A maximal biomass of 15.5 ± 0.9/gL was obtained at the end of the culture, achieving around a 49% higher biomass level following the addition of methanol (Table 1).

A comparison between the BSFs and bioreactor cultures showed there to be similar specific growth rates during the *P. pastoris* X-33 (rPstS-1) batch growth phase in the glycerol containing culture medium, which is similar to previously reported results [84, 85]. However, during the methanol induction phase, the specific growth rate double in the bioreactors compared to the BSFs. Additionally, the maximal biomass reached in the bioreactors was higher in both BMGY and BMMY, being around 19 and 43% higher, respectively, compared to that in the BSFs (Table 1). This might be caused by the limited oxygen transfer in the BSFs compared to the bioreactor cultures [86–88], thereby affecting the post-induction specific growth rate [83]. Differences in biomass/glycerol yield were also observed, with a 17% lower in the BSFs compared to the bioreactor cultures. Moreover, the yields obtained in this study are in agreement with those reported by Chiruvolu et al. [89], who reported values between 0.70 and 0.78 g_DCW_/g_Glyc_ for bioreactors without pH control and 0.62 g_DCW_/g_Glyc_ in shake flasks. No significant differences were observed in glycerol consumption rate (q_s_) between the bioreactor and BSF cultures.

### Production of rPstS-1, its characterization by SDS-PAGE, and immunodetection

**Fig. 2:**
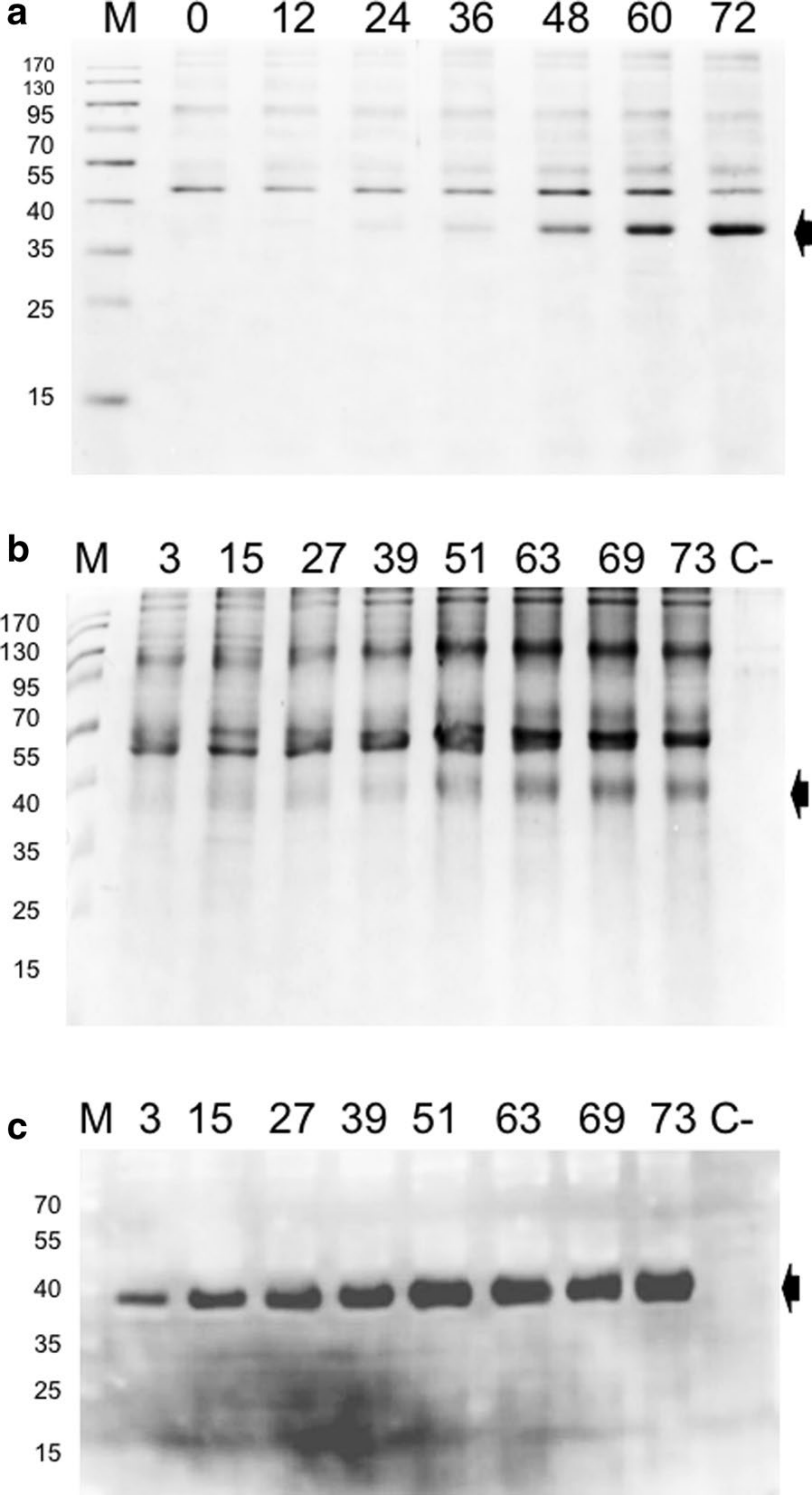
Total protein analysis from culture supernatant. **a** SDS-PAGE 12% of protein obtained from baffled shake flask cultures, **b** SDS-PAGE 12% of protein obtained from bioreactor supernatants. Black arrows indicate the band corresponding to rPstS-1. **c** Immunodetection of rPstS-1 from bioreactor supernatants with the polyclonal antibody anti-PstS1, black arrows indicate the immunodetected recombinant protein. The post-induction time for each sample taken is indicated at the top of each lane. M, molecular weight; C−, negative control; supernatant sample from a wild type *P. pastoris* X-33 culture

At the end of the BSFs culture, the total protein content of the supernatant was 127 ± 15 mg/L. A densitometric analysis of the secreted proteins by SDS-PAGE (Fig. 2a) showed that the rPstS-1 protein (~ 38 kDa) represented ~ 36% (46 ± 5 mg/L, Table 1) of the total secreted protein. After the induction in the BSFs, relatively low-levels of extracellular host proteins were detected (Fig. 2a, time 0 post-induction), which is one advantage of producing recombinant proteins in *P. pastoris* [66, 90].

After 72 h of induction with methanol in the bioreactor cultures, the total protein content of the supernatant was 185 ± 9 mg/L (Table 1). The level of secreted rPstS-1 in the supernatant was 46 ± 4 mg/L (Fig. 2b, Table 1). To confirm the identity of rPstS-1, the supernatant was analyzed by a western blot assay using a polyclonal antibody anti-pstS1 (Fig. 2c). The 38 kDa protein was confirmed to be rPstS-1 and interestingly, there were no apparent sign of protein degradation, nor was rPstS-1 detected in the supernatant before methanol induction (Fig. 2c).

It is important to note that although there was a 69% higher level of total protein in the supernatant produced in the bioreactors compared to the BSFs, the absolute levels of rPstS-1 were similar (46 ± 5 and 46 ± 4 mg/L, in the BSFs and bioreactor, respectively). As a result, there was a decrease in the specific production rate of rPstS-1 in the bioreactors (32% lower than the BSFs, Table 1), while the volumetric production rate of rPstS-1 in both systems was the same (Table 1). The level of accumulation of the folded rPstS-1 protein in *P. pastoris* was almost three times higher per liter compared to previous published bacterial system [91].

### Purification of rPstS-1 from supernatants

**Fig. 3:**
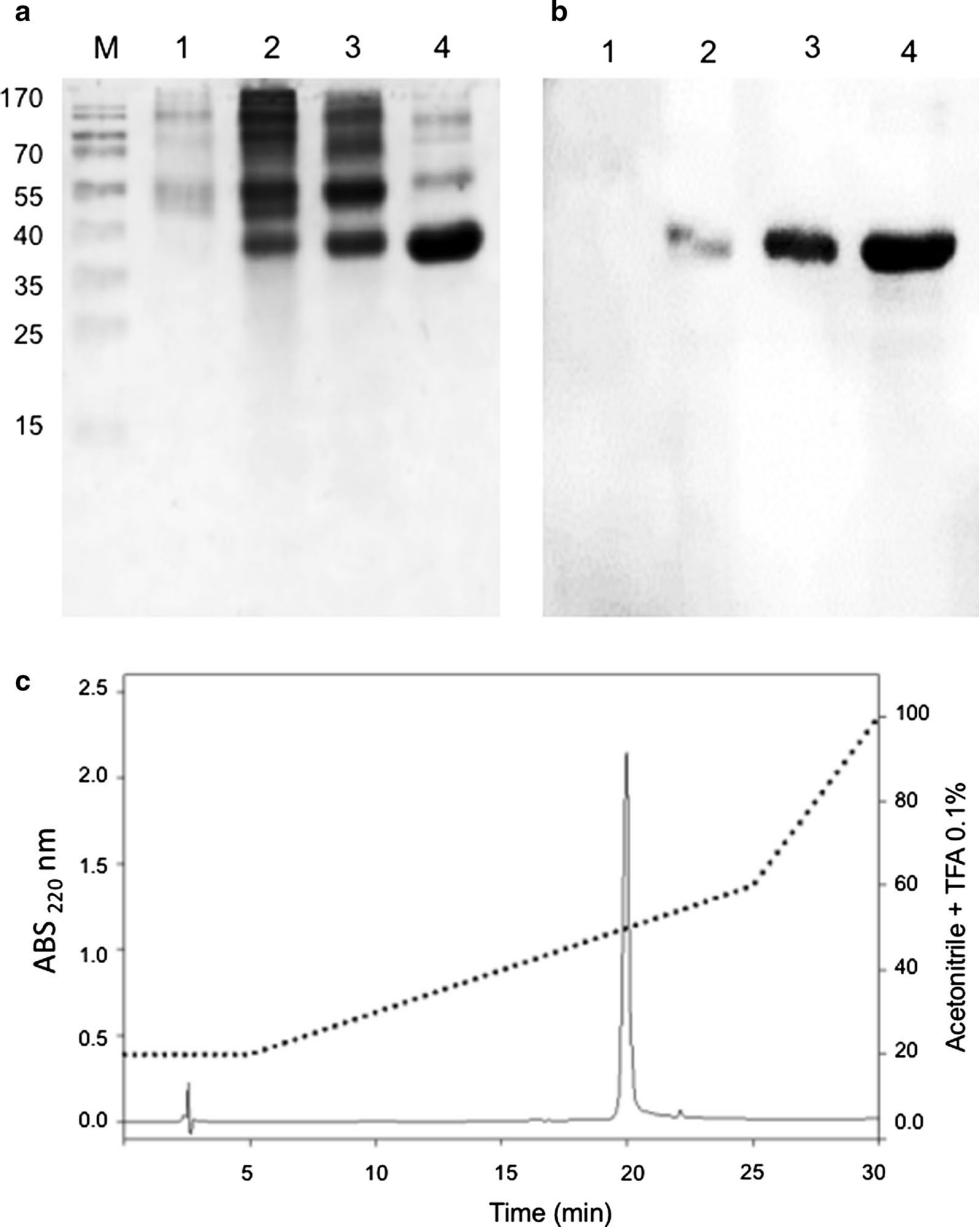
Purification of rPstS-1. **a** SDS-PAGE 12% during purification process. **b** Immunodetection of protein obtained by ultrafiltration and RP-HPLC. **c** Separation of components obtained after tangential filtration in a XBridge Protein BEH C4 reverse-phase column, from which the component eluting at 20 min was shown to be homogeneous. The gradient started by 5 min at 20% of B, then separation was run from 20 to 60% solution B, over next 20 min, and the pure rPstS-1 eluted at 20.74 min. Lanes M: molecular weight marker; 1, total protein from supernatant of the 27 h growth in BMGY; 2, total protein from supernatant obtained after 72 h under induction; 3, 10–100 kDa protein cut off from tangential filtration; 4, protein purify by RP-HPLC

Purification of rPstS-1 from the *P. pastoris* bioreactor culture supernatant was carried out by ultrafiltration, which is often used as an alternative method to the affinity chromatography procedures used for tagged proteins and does not require any refolding steps, in comparison with other bioprocesses [91, 92]. Thus, rPstS-1 was purified in a three-step procedure: first, the culture was clarified by tangential filtration (100 kDa); then, the supernatant was subjected to another tangential filtration (10 kDa); and finally, RP-HPLC was used. The two tangential filtrations gave recoveries of 41% and 29%, respectively (Table 2). After the RP-HPLC step, a recovery of 4 mg/L of rPstS-1, with a purity of 98% was obtained, with a retention time of 20 min, eluting in H_2_O:ACN:TFA (50:50:0.1) (Fig. 3c). Furthermore, the purification steps were monitored by SDS-PAGE (Fig. 3a), and the densitometric analysis of SDS-PAGE showed a purity of up to 90% for rPstS-1 after the final step (Fig. 3a, line 4). Also, the presence of rPstS-1 during the different purification steps was followed by western blot (Fig. 3b). This study clearly demonstrates the utility of tangential filtration technology to purify *O*-mannosylated rPstS-1 without affinity tags, from a *P. pastoris* culture supernatant. It should be noted that the technology presented here is scalable and can be applied for the large-scale production of rPstS-1 and other *Mtb* antigens produced in a similar fashion.

**Table 2:** Summary of purification of rPstS-1

Purification stage	Recovery of total protein (mg/L)^a^	rPsts-1 (mg/L)^a^	Recovery %	Purity %
Total protein from supernatant	185 ± 9	46 ± 4	100	25 ± 3
Ultrafiltration (> 100 kDa)	80.0 ± 2.9	18.9 ± 5.0	41.0 ± 14.9	23.5 ± 5.4
Ultrafiltration (100–10 kDa)	49.0 ± 3.4	13.2 ± 4.2	28.6 ± 7.1	26.5 ± 6.7
Reverse phase chromatography	4.3 ± 0.2	4.2 ± 0.2	9.1 ± 1.1	97.7 ± 0.6

^a^Total supernatant protein was calculated by Bradford method. The rPstS-1 protein was calculated by densitometry in gel after each stage of purification, and each protein purification step was calculated per liter of initial cell culture volume

### Structural conformation of rPstS-1

**Fig. 4:**
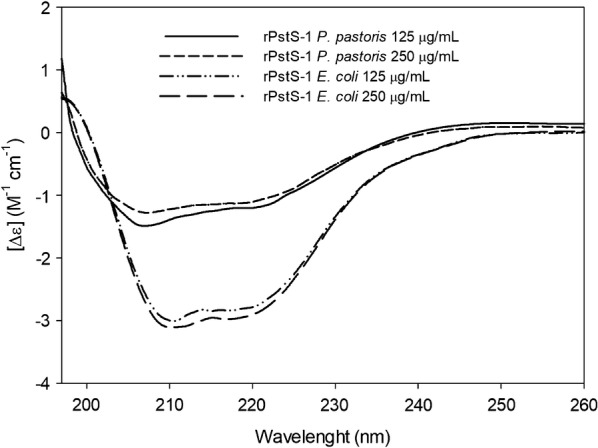
Structural analysis of rPstS-1 from *P. pastoris* and *E. coli* purified by HPLC by Circular dichroism spectrum scanned over 190–260 nm range at 125 and 250 μg/mL

The secondary structure of rPstS-1 was determined using far-UV circular dichroism (CD) spectroscopy. At both the rPstS-1 concentrations tested, the spectra obtained at 25 °C showed a maximum negative peak at 207 nm, in addition to a broad peak between 212 and 219 nm, and a positive peak at 197 nm, (Fig. 4). A molar ellipticity of − 4916.67 deg*cm^2^*/dmol was obtained for rPstS-1 at 0.125 mg/mL and − 4209.29 deg*cm^2^*/dmol for rPstS-1 at 0.250 mg/mL concentration. Analysis of the CD spectrum of rPstS-1 using CAPITO [93] showed a secondary structure consisting of 10% α-helix, 38% beta sheets, and 56% disordered structures for rPstS-1 at 0.125 mg/mL. Moreover, at 0.250 mg/mL rPstS-1 the spectrum showed 5% α-helix, 40% β-sheets, and 53% disordered structures (Fig. 4, Table 3). The high content of β-sheets is in agreement with the CD spectrum of the purified native denaturated and refolded 38 kDa protein obtained from a H37Rv culture, which showed a negative broad peak at 212 nm and a positive peak at 190 nm, suggesting a high β-sheet content [12].

**Table 3:** Secondary structure of rPstS-1 from *P. pastoris* and *E. coli*. Comparative secondary structure content obtained by CD data analysis, NMR data and CAPITO server

	NMR [94]	CAPITO (CD)			
		rPstS-1 from *E. coli*		rPstS-1 from *P. pastoris*	
	PDB: 1PC3	125 μg/mL	250 μg/mL	125 μg/mL	250 μg/mL
Helix (%)	34	16	20	10	5
β-strand (%)	18	28	29	38	40
Irregular (%)	41	50	52	56	53

*CAPITO* CD Analysis & Plotting Tool [93], *CD* circular dichroism, *NMR*, nuclear magnetic resonance; *PDB* protein data bank

On the other hand, the spectra of rPstS-1 produced in *E. coli* showed a maximum negative peak at 209 nm with a broad peak around 218 nm at both protein concentrations (Fig. 4). The rPstS-1 expressed in *E. coli* showed an increased α-helix content (16% α-helix at 125 μg/mL and 20% α-helix at 250 μg/mL, Fig. 4, Table 3).

The data obtained in this study show peaks that are similar to those in the CD spectrum obtained from the recombinant 38 KDa reference antigen from the WHO bank, which has negative peaks at 208 nm and 225 nm [12], and also the spectrum from the full length rPstS-1 expressed in *E. coli* which also shows negative peaks at 208 nm and 218 nm, in addition to a positive peak at 190 nm [92]. However, an analysis of the rPstS-1 secondary structure using CDNN software showed that rPstS-1 expressed in *E. coli* had a conformation consisting of 40% α-helices and 8.9% antiparallel and 5.6% parallel β-sheets [93]. In addition, the data obtained from the X-ray structure of rPstS-1 expressed in *E. coli* showed a composition of 34% α-helices, 18% beta sheets, and 41% disordered structures (PDB accession No. 1PC3) [94]. The CD spectrum of rPstS-1 produced in *E. coli* points to a predominant α-helix peak (208 nm), which differs from the secondary structure suggested by the spectrum of the purified, denaturated, and refolded native 38 kDa antigen from H37Rv [12]. These differences might arise due to the tags added, the purification steps used, or the refolding process used for proteins expressed in *E. coli*. The data obtained in this study suggest that the secondary structure of rPstS-1 produced in *P. pastoris* differs from that produced in bacteria. Moreover, the rPstS-1 produced here has a β-sheet conformation that agrees with that observed for the native protein [12]. This is probably related to the *O*-mannosylation of rPstS-1, which is similar to the native protein expressed in *M. tuberculosis* which has approximately a 1% carbohydrate content [36]. Moreover, the rPstS-1 produced in *P. pastoris* was in a soluble form, thereby avoiding the need for a refolding step, which is needed for many proteins produced in *E. coli.* An understanding of how *O*-mannosylation affects the protein conformation is important to establish whether this modification is an important parameter for the correct folding of a protein.

### Mass spectrometry analysis of the rPstS-1 antigen

**Fig. 5:**
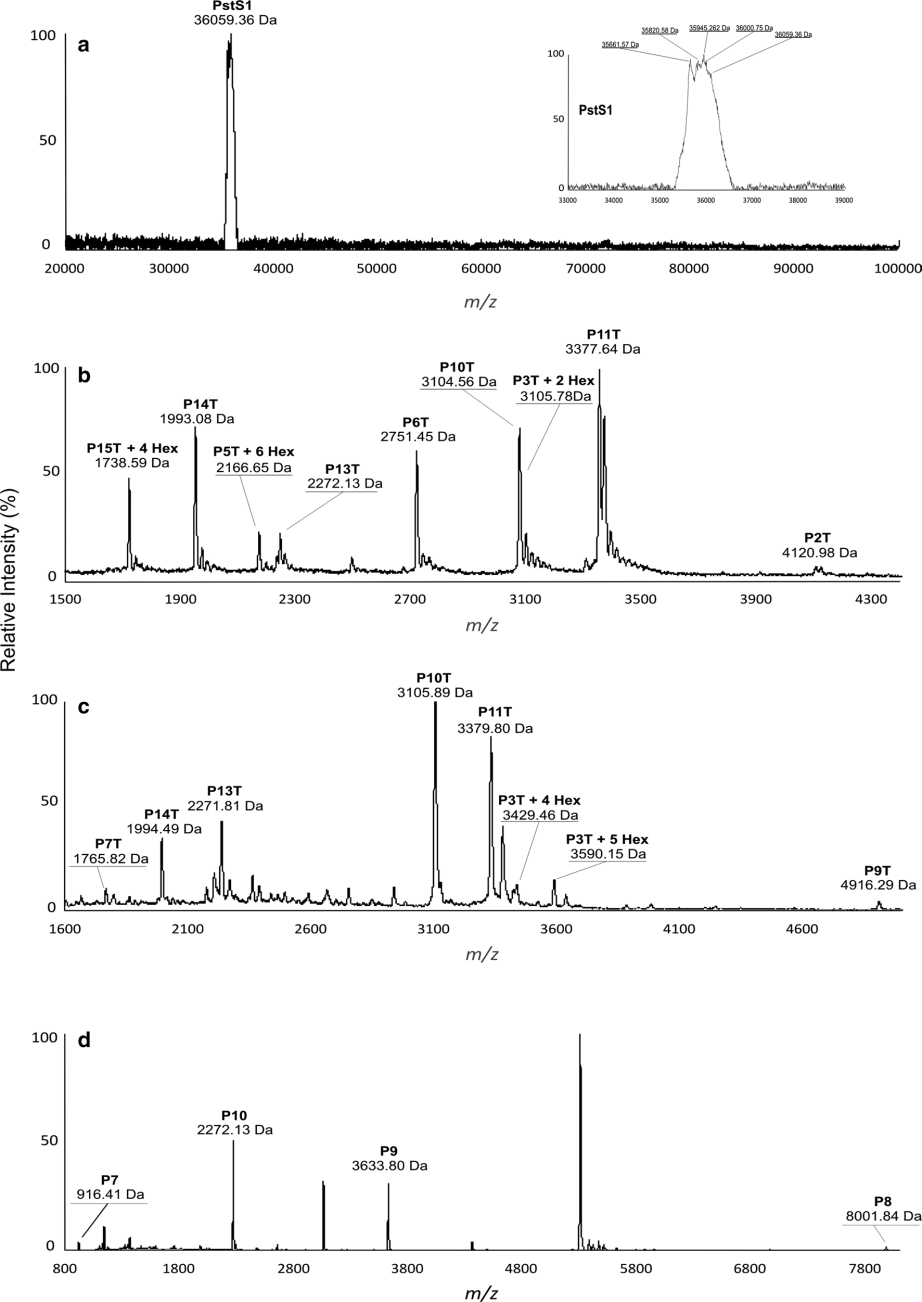
MALDI-TOF spectra of HPLC purified rPstS-1 antigen. **a** Non-digested samples were placed on a CHCA (α-Cyano-4-hydroxycinnamic acid) matrix and analyzed in a matrix assisted laser desorption ionization time-of-flight (MALDI-TOF, Bruker Microflex) equipment with a 20-Hz nitrogen laser at I = 337 nm. Spectra was recorded in linear positive mode for the mass range 20,000 to 100,000 Da. **b** MALDI-TOF–MS analysis (linear mode) of a tryptic digest of rPstS-1, sample mixed with α-cyano-4-hydroxycinnamic acid matrix. **c** MALDI-TOF–MS analysis (linear mode) of a tryptic digest of rPstS-1, sample mixed with α-cyano-4-hydroxycinnamic acid and 3,5-dimethoxy-4-hydroxycinnamic acid matrices. **d** MALDI-TOF–MS analysis (linear mode) of a Lys-C digest of rPstS-1. Tryptic and Lys-C peptides marked denote mass peaks corresponding to predicted peptides shown in Tables 4 and 5, respectively. Mannose residues were detected with differences to theoretical and experimental data of 162 Da mark as Hex. All detections are presented in Table 4 and 5

The full amino acid sequence of the PstS-1 gene product deduced from the sequence of the *Mtb* consists of 374 residues, with a theoretical MW of 38,243 Da (UniProtKB P9WGU1). The first 23 amino acid residues form a signal peptide, which directs lipidation at the N-terminus [37]. In this study, rPstS-1 was produced without the first 21 amino acids to avoid lipidation, so the recombinant protein had a predicted MW of 36,059.36 Da, and this in agreement with the peak shown in Fig. 5a. A zoomed view of the peak (Fig. 5a inset) shows a family of different isoforms, probably arising to different post-translational modifications (PTMs), that might correspond to the attachment of hexose units, along with other types of modifications. It is worth mentioning that in the native PstS-1, the *O*-mannosylation pattern sites and the number of mannose units have not been determined. A prediction program (Glycopp v1.0) [95] for prokaryotic glycosides indicated that there were 26 possible *O*-glycosylation sites in rPstS-1, with nine sites being localized at the N-terminus (in the first 25 amino acids).

In order to analyze the rPstS-1 amino acid sequence and *O*-mannosylation, rPstS-1 was digested with the enzymes Lys-C or trypsin. The resulting peptides were analyzed by matrix assisted laser desorption ionization time-of-flight (MALDI-TOF) spectrometry. The theoretical fragment profile expected for trypsin or Lys-C digestion and the results obtained are shown in Tables 4 and 5, respectively. The peaks obtained from the digestions covered 83% of the sequence. These results indicate that the amino acids comprising to positions 55 to 97 and 138 to 342 were not modified by the addition of mannose residues. Around 20% of the peptide sequence obtained by MS/MS is presented in Additional file 1: Figure S1. In contrast, the N-terminal sequence formed by the first 97 amino acids (P1T) was not detected in either the peptides formed by trypsin digestion (P4T, P8T and P12T), or in those formed by Lys-C digestion (P1, P2, P3, P4, P5, P6, P11, and P12) (Table 5). This could probably be due to a lack of ionization, the inherent hydrophobicity of the peptides, or the presence of hexose residues.

**Table 4:** Theoretical and obtained peptides mass generated by Trypsin digestion of rPstS-1 produced in *P. pastoris*

Peptide name	Position of cleavage site	Peptide	Length (aa)	Theoretical Peptide mass (Da)	Modification	Peptide mass (Da)
P1T	54	AGCGSKPPSGSPETGAGAGTVATTPASSPVTLA ETGSTLLYPLFNLWGPAFHER	54	5315.900		ND
P2T	97	YPQVTITAQGTGSGAGIAQAAAGTVNIGASDAYL SEGDMAAHK	43	4122.489		B) 4120.98
P3T	123	GLMNIALAISAQQVNYNLPGVSEHLK	26	2781.222 +2 Hex 3105.22 +4 Hex 3429.22 +5 Hex 3591.22	P3T + 2 Hex P3T + 4 Hex P3T + 5 Hex	B) 3105.78 C) 3429.46 C) 3590.15
P4T	127	LNGK	4	430.504		ND
P5T	138	VLAAMYQGTIK	11	1194.455	P5T + 6 Hex	B) 2166.65
P6T	164	TWDDPQIAALNPGVNLPGTAVVPLHR	26	2752.125		B) 2751.45
P7T	180	SDGSGDTFLFTQYLSK	16	1765.894		C) 1765.82
P8T	188	QDPEGWGK	8	915.958		ND
P9T	238	SPGFGTTVDFPAVPGALGENGNGGMVTGCAE TPGCVAYIGISFLDQASQR	50	4918.459		C) 4916.29
P10T	270	GLGEAQLGQSSGNFLLPDAQSIQAAAAGFASK	32	3105.411		B) 3104.56 C) 3105.89
P11T	301	TPANQAISMIDGPAPDGYPIINYEYAIVNNR	31	3378.761		B) 3377.64 C) 3379.80
P12T	303	QK	2	274.320		ND
P13T	324	DAATAQTLQAFLHWAITDGNK	21	2272.502		B) 2272.13 C) 2271.81
P14T	342	ASFLDQVHFQPLPPAVVK	18	1993.336		B) 1993.08 C) 1994.49
P15T	353	LSDALIATISS	11	1090.239	P15T + 4 Hex	B) 1738.59

Mark of B) and C) referred the mass spectra from Fig. 4b and c, respectively

*Hex* hexose, *ND* no determined, *T* trypsin, *aa* amino acids

**Table 5:** Theoretical and obtained peptides mass generated by Lys-C digestion of rPstS-1 produced in *P. pastoris*

Peptide name	Position of cleavage site	Peptide	Length (aa)	Theoretical Peptide mass (Da)	Modification	Peptide mass (Da)
P1	6	AGCGSK	6	521.589		ND
P2	97	PPSGSPETGAGAGTVATTPASSPVTLAETGSTL LYPLFNLWGPAFHERYPQVTITAQGTGSGAGIA QAAAGTVNIGASDAYLSEGDMAAHK	91	8916.801		ND
P3	123	GLMNIALAISAQQVNYNLPGVSEHLK	26	2781.222		ND
P4	127	LNGK	4	430.504		ND
P5	138	VLAAMYQGTIK	11	1194.455		ND
P6	180	TWDDPQIAALNPGVNLPGTAVVPLHRSDGSGD TFLFTQYLSK	42	4500.004		ND
P7	188	QDPEGWGK	8	915.958		D) 916.41
P8	270	SPGFGTTVDFPAVPGALGENGNGGMVTGCAE TPGCVAYIGISFLDQASQRGLGEAQLGQSSGN FLLPDAQSIQAAAAGFASK	82	8005.854		D) 8001.84
P9	303	TPANQAISMIDGPAPDGYPIINYEYAIVNNRQK	33	3635.065		D) 3633.80
P10	324	DAATAQTLQAFLHWAITDGNK	21	2272.502		D) 2272.13
P11	342	ASFLDQVHFQPLPPAVVK	18	1993.336		ND
P12	353	LSDALIATISS	11	1090.239		ND

Mark of D) referred the mass spectrum from Fig. 4d

*Hex* hexose, *ND* no determined

The peptide that was found to be mannosylated was P3T (aa. 98–123: GLMNIALAISAQQVNYNLPGVSEHLK) which is located near the N-terminus, with 2, 4, and 5 mannose residues indicated by the addition of 162 Da per hexose (Fig. 5b, c; Tables 4, 5). In addition, P5T (aa. 128–138: VLAAMYQGTIK) was also found to have 6 hexose residues (Fig. 5b); whereas, P15T at the C-terminus (aa. 343–353: LSDALIATISS) was found to have 4 hexose residues attached (Fig. 5b). These three peptides contained serine or threonine residues (underlined), in accordance with them containing potential *O*-glycosylation sites (regions rich in S or T) as has been proposed to occur in *S. cerevisiae.* Attachment of the mannose residues appears to be performed in part by the protein-*O*-mannosyltransferase (PMT), which is conserved in yeasts and is similar to the enzymes found in *P. pastoris* [96, 97].

### rPsts-1 sero-reactivity with human sera samples

**Fig. 6:**
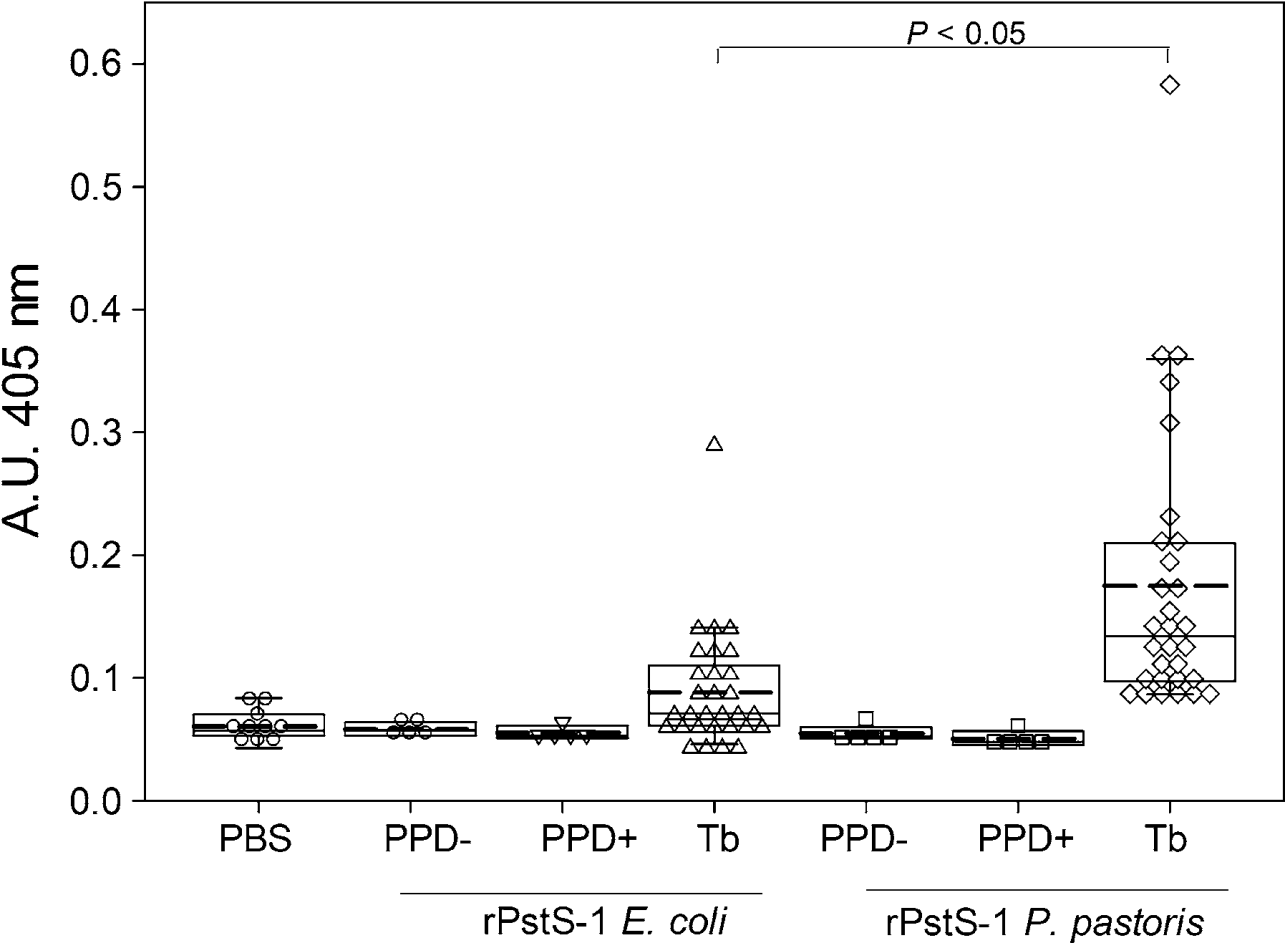
Box plot analysis of antibody reactivity to recombinant antigens rPstS-1. Recombinant proteins (from *E. coli* and *P. pastoris*) were tested with PPD-negative (n = 5), PPD-positive sera (n = 5) at 1:100 and active-TB sera (n = 30) at 1:500 dilution. The reactivity is reported as difference of the absorbance units observed for the different group of sera by ELISA using a IgG2 conjugated with AP. Different sera of each group were tested by duplicate. Mean (dashed lane), median, 10th, 25th, 75th and 90th percentiles as vertical boxes with error bars are shown. Dunn´s test ws used for all pairwise comparisons and comparisons against a control group (PBS) following rank-based ANOVA

With the intention of confirming the similarity of rPstS-1 to the 38 kDa native antigen, the sero-reactivity of rPstS-1 was assessed using an ELISA method. This was carried out using human serum samples from patients with clinically confirmed active TB, and compared with sera from subjects with both positive and negative PPD test, which did not present any clinical evidence of active TB. Thirty sera from patients with active TB (these characteristics are described in methods) showed a statistically positive response against the rPstS-1 produced in *P. pastoris*, compared with the positive and negative PPD groups (Fig. 6). To confirm the significance of the glycosylation pattern of rPstS-1 produced in *P. pastoris*, the sero-reactivity of the non-glycosylated rPstS-1 version produced by *E. coli* cultures [23] was also determined. The recognition of the rPstS-1 from *P. pastoris* by serum from IgG2 in patients with active tuberculosis were 3.2 and 3.5 -fold higher (mean values) than the PPD-negative and PPD-positive subjects, respectively (Fig. 6). Importantly, rPstS-1 produced in *P. pastoris* showed a statistically significant (*P *< 0.05) 2.0-fold (mean) higher recognition compared with the non-glycosylated His-tag-rPstS-1 produced in *E. coli* (Fig. 6). Recognition by IgG2 was used in this study, because it is known that it recognizes carbohydrates and protein antigens from bacteria [98, 99]. The recognition of *P. pastoris* rPstS-1 by sera from patients with TB suggested that the recombinant protein has a conformation close to the native protein. This might be related to the *O*-mannosylation of rPstS-1 that could improve recognition by TB serum compared to the recognition of the non-glycosylated rPstS-1 produced in *E. coli*. In other previously reported sero-reactivity experiments the detection of the rPstS-1 produced in *E. coli*, by antibodies derived from patients with TB varied from 36 to 77% [92, 100, 101], probably due to the lack of glycosylation of the protein.

Up until now, different diagnostic kits such as the PPD skin test or the QuantiFERON^®^-TB test have been developed, which are T cell based immune assays, and they do not distinguish between latent and active tuberculosis. The development of sero-diagnostic test using secreted protein antigens derived from *Mtb*, such as PstS-1, has been proposed. This is because these antigens are recognized by the TB patients’ antibodies with high sensitivity and so this should aid in the development of sensitive diagnostics to distinguish between the inactive and active TB states [101–103].

To qualitatively estimate the antibody avidities for *P. pastoris* rPstS-1 and *E. coli* expressed His-tag-rPstS-1, we carried out western blot experiments [64] using a rabbit anti-*Mycobacterium tuberculosis*-PstS-1 polyclonal antibody and a human serum sample from a patient with clinically confirmed active TB. Additional file 2: Figure S2A shows that the anti-PstS-1 polyclonal antibody reacted strongly towards the *P. pastoris* rPstS-1 compared with the *E. coli* expressed His-Tag-rPstS-1. The relative band intensities (measured by densitometry), of 2.0 and 1.0 μg of *P. pastoris* rPstS-1 appeared to be 3.9 and 5.1 times higher than *E. coli* His-tag-rPstS-1, respectively, suggesting an increased immunoreactivity toward the *O*-mannosylated rPstS-1.

On the other hand, the relative band intensities for the human serum reactivity toward 2.0 and 1.0 μg of *P. pastoris* rPstS-1 appeared to be 2.5 and 1.2 times higher than *E. coli* His-tag-rPstS-1, respectively (note the band of around 38 KDa, Additional file 2: Figure S2B). Although, recognition of a protein with a high molecular weight in the *P. pastoris* rPstS-1 sample was observed, in the 2.0 μg *E. coli* rPstS-1 sample a protein of around 10 KDa was also detected, indicating there is low cross-reactivity between the experiments. The higher detection of *P. pastoris* rPstS-1 compared with *E. coli* rPstS-1 is probably due to posttranslational differences in the *P. pastoris* produced protein.

## Conclusions

In this work, using *Pichia pastoris* as the heterologous host, we achieved the synthesis and production of an untagged recombinant *O*-mannosylated rPstS-1 antigen, containing all the predicted B-cell epitopes. Under the BSF and bioreactor culture conditions tested, we were able to achieve an accumulation of around 46.2 ± 4.4 mg/L, which is three times more protein per liter compared to bacterial systems. Furthermore, we have shown the recovery and purification of rPstS-1 using a simple and efficient procedure, finally obtaining 4 mg/L rPstS-1 with 98% purity, after three purification steps, and without any need for a refolding step. The circular dichroism spectra of rPstS-1 produced in *P. pastoris* showed that there were differences with the structure reported for this protein produced in bacteria; the *O*-mannosylated rPstS-1 here had a high β-sheet content similar to the purified native 38 kDa PstS-1 recovered from a culture filtrate from *M. tuberculosis* H37Rv. Moreover, the conformational similarity of the *O*-mannosylated rPstS-1 produced to that of the native antigen (38 kDa), was confirmed indirectly by its ability to be recognized by antibodies derived from the sera of patients positive for tuberculosis, compared to the purified non-glycosylated version (His-tag-rPstS-1) produced in *E. coli*. The detection of *O*-mannosylated rPstS-1 by active TB serum, emphasizes the potential utility of this antigen for the development of a sensitive sero-diagnostic kit for TB detection using the *P. pastoris* recombinant system.

## Methods

### Reagents and strains

For culture media components, peptone, yeast extract, and agar were purchased from Difco™ (Becton–Dickinson, Franklin Lakes, NJ, USA), methanol, peptone, dextrose, and salts were from JT Baker (Phillipsburg, NJ, USA), and sorbitol and Triton X-100 were obtained from Merck (Billerica, MA, USA). For molecular biology reagents, Zeocin™, T4 ligase, and PCR reagents were purchased from Thermo Fisher Scientific (Waltham, MA, USA), the PstI and KpnI restriction enzymes were purchased from Jena Bioscience GmbH (Jena, Germany), and the plasmid purification kit Zyppy™ Plasmid Miniprep Kit was bought from Zymo Research (Irvine, CA, USA). Protein precipitation, SDS-PAGE and western blotting reagents were purchased from Merck, and the Super Signal^®^ West Pico Chemiluminescent substrate was purchased from ThermoFisher Scientific. The HPLC grade reagents acetonitrile and trifluoroacetic acid were purchased from JT Baker (Phillipsburg, NJ, USA). Plasmid propagation and subcloning steps were carried out in One Shot™ TOP 10 chemically competent *E. coli* purchased from ThermoFisher Scientific and cultured in Luria–Bertani medium (Merck). The expression of rPstS-1 was carried out in *Pichia pastoris* X-33 using the pPICZαB vector (ThermoFisher Scientific). *E. coli* His-tagged-rPstS-1 was expressed as previously described [23].

### Vector construction: pPICZαB-PstS1

The gene sequence encoding PstS1 (Rv0934) was obtained from GenBank ID No. 885724. This nucleotide sequence was then modified to create the preferential codon usage for *P. pastoris* [70, 71]. To avoid N-glycosylation, the asparagines at residues 57 and 247 were substituted with glutamines [79]. The optimized sequence was chemically synthesized and cloned into the pUC57 plasmid by GenScript (Piscataway, NJ. USA). The recombinant vector pPICZαB-PstS-1 was obtained from ligation of the chemically synthesized gene, previously digested with PstI and KpnI, to the pPICZαB vector using T4 DNA ligase. The fragment was inserted into pPICZαB downstream of the α-mating factor secretion signal and under the control of the AOX1 (alcohol oxidase 1) promoter. A stop codon (TAA) was incorporated at the 3′ end of the sequence. The recombinant plasmid pPICZαB-PstS-1 containing the coding sequence for the non-tagged PstS-1 was then propagated using One Shot™ TOP 10 chemically competent *E. coli* cultured in Luria–Bertani medium containing 25 μg/mL Zeocin™. The plasmid was purified and the sequence of the insert determined. The resulting pPICZαB-PstS-1 plasmid was linearized and used to transform electro-competent *P. pastoris* X-33 suspended in 1 M sorbitol, at a setting of 1.5 kV, 25 μF, and 186 ohms for the BTX Electroporation System (Genetronics, San Diego, CA, USA). Transformed cells were selected using YPD agar (yeast extract 10 g/L, dextrose 20 g/L, peptone 20 g/L, 1 M sorbitol, and agar 20 g/L) supplemented with Zeocin™ (100 μg/mL) at 30 °C. Positive clones were then stored in 1.0 mL cryovials at − 80 °C in 20% glycerol. The clones producing the highest protein expression levels of rPstS-1 were selected using SDS-PAGE.

### Analytical methods

*Pichia pastoris* growth was determined by measuring the OD_600_ (Spectronic Genesys 20, Thermo Fisher Scientific). Biomass was evaluated by measuring dry weight; 5 mL of culture was filtered through a 0.45 μm pore size membrane (Merck-Millipore, Billerica, MA, USA), and washed once with one volume of distilled water. The biomass obtained was dried for 24 h in an oven at 65 °C, then placed for 1 h in a desiccator, and weighed thereafter. The supernatant was used to measure glycerol consumption using a colorimetric method previously described by Müller et al. [104]. Methanol consumption was determined using the Biochemistry Analyzer YSI 2900 (YSI Life Sciences, Yellow Springs, OH, USA).

### Production of *P. pastoris* rPstS-1 in shaker flasks and bioreactors

Baffled shake flasks (BSF, 250 mL, Duran^®^ Erlenmeyer flask, narrow neck, Borosilicate Glass, Mainz, Germany) containing 50 mL of BMGY media (yeast extract 10 g/L, peptone 20 g/L, glycerol 10 mL/L, and 100 mM potassium phosphate, initial pH of 6.0) were inoculated with 200 μL of culture from cryovials of the selected clone. After the glycerol was depleted, the cells were harvested by centrifugation at 6300×*g* for 15 min and the resulting pellet was resuspended in 50 mL of BMMY (yeast extract 10 g/L, peptone 20 g/L, methanol 10 mL/L, and 100 mM potassium phosphate, pH 6.0). All cultures were incubated at 30 °C in an orbital shaker at 250 rpm (New Brunswick Scientific C251, Eppendorf Inc., Enfield, CT, USA).

For bioreactor experiments (containing 1.0 L of BMGY medium, Applikon Biotechnology, Netherlands), the medium was inoculated with BSF cultures at an initial OD_600_ of 0.5 AU. The BSF inoculum was derived by inoculating 50 mL of YPD with one vial of the selected clone and incubating at 30 °C and 250 rpm for 24 h. Bioreactor cultures were carried out at 29 °C, without pH control and the dissolved oxygen tension (DOT) was set at 35% (with respect to air saturation) and controlled by cascade, changing the agitation speed (between 200 and 1000 rpm), maintaining an airflow of 1.0 L/min (1 vvm) using a proportional-integral-derivative (PID) control strategy [105]. DOT, temperature, agitation and pH values were acquired and controlled by the ADI-1010 and BioXpert^®^ software (Applikon Biotechnology). The total consumption of glycerol (12.6 g/L) was assumed to have occurred when 95% of the DOT was reached, at which point the induction phase was started by the addition of methanol (7.9 g/L). Each time the DOT reached above 80%, the feeding of methanol (7.9 g/L) was done. Samples were collected every 6 h (1.0 mL), the supernatants were obtained by centrifugation at 8000×*g*, and frozen for further analysis. All cultures were grown as three independent replicates and the results are expressed as the mean ± standard deviation.

### Production of *E. coli* rPstS-1 in bioreactors

The *Escherichia coli* (strain DE3) used to produce rPstS-1 was grown in a bioreactor (1.0 L Luria–Bertani culture medium, Applikon Biotechnology). The bioreactor cultures were carried out at 37 °C, without pH control, and the DOT was set at 35% (with respect to air saturation) and controlled by cascade changing the agitation speed (between 200 and 1000 rpm), maintaining an airflow of 1.0 L/min (1 vvm) using a proportional-integral-derivative (PID) control strategy [105]. After 4 h of growth (OD_600_ near 1.5 A.U.) the induction phase was started by the addition of 0.1 mM of isopropyl-β-D-thiogalactopyranoside (IPTG). After 4 h of induction (O.D._600_ of 5.9 AU), the biomass was recovered by centrifugation. The cell suspension was sonicated in lysis buffer containing 0.5% (v/v) Triton X-100 and the samples were centrifuged (10,000×*g* for 10 min). The recombinant PstS-1 was produced in inclusion bodies, which were solubilized using a denaturation buffer (100 mM NaCl, 50 mM Tris–HCl, 6 M GuHCl, pH 8).

### Total protein quantification, SDS-PAGE, and western blotting

The concentration of total soluble proteins in the supernatants was determined using a Bradford protein assay (Bio-Rad Inc, Hercules, CA, USA) following the supplier’s recommendations. Calibration curves were prepared using bovine serum albumin. Samples and standards were prepared as triplicates and absorbances measured at 595 nm using a plate reader (Stat Fax 4200, Awareness Technology, Palm City, FL, USA). Total protein from 500 μL of supernatant was precipitated with 1 volume of trichloroacetic acid (30%) in cold acetone. Precipitation was carried out overnight at − 20 °C followed by centrifugation at 14,000×*g* for 25 min. Pellets were washed with acetone (80%), dried at 60 °C for 5 min, and then were suspended in 20 μL of Milli-Q water. The precipitated samples were separated by electrophoresis on 12% SDS-PAGE gels [106]. Proteins were visualized by staining with Coomassie Brilliant Blue R-250 the stained gels imaged using Image-LabTM software and the Gel DocTM EZ System (Bio-Rad Inc, USA). A pre-stained protein ladder PageRuler™ (Thermo Fisher Scientific, USA) was used to assess molecular mass.

To detect recombinant proteins by western blot, the proteins were separated by SDS-PAGE and then transferred to a polyvinylidene difluoride (PVDF) Immobilon-P™ membrane. The transblotted membranes were blocked with skimmed milk (5%) in phosphate-buffered saline (PBS) containing Tween-20 (0.05%). After three washes with PBS-Tween-20, the membranes were incubated with an anti-*Mycobacterium tuberculosis*-PstS-1 polyclonal antibody (ArtNr: OACA02044. Aviva System Biology Co., San Diego, CA, USA; diluted to 1/2000) for 1 h at room temperature. After incubation, the membranes were washed twice with PBS-Tween 20 and incubated with peroxidase conjugated anti-mouse immunoglobulin G antibodies diluted 1/2000, at room temperature for 30 min. Membranes were again washed twice with PBS-Tween, and the immunoreactive bands revealed by chemiluminescence (Thermo Scientific, SuperSignal^®^ West Pico Chemiluminescent Substrate). The bands were visualized using a C-digital scanner (LI-COR, NE, USA).

For a comparative study of the ability of the antigens to be recognized by an antibody, western blot experiments were performed [64] using three different amounts (2, 1, and 0.5 μg) of rPstS-1 produced in either *P. pastoris* or *E. coli*. The membrane was incubated with a rabbit anti-*Mycobacterium tuberculosis*-PstS-1 polyclonal antibody (ArtNr: OACA02044. Aviva System Biology Co. USA; diluted 1/2000) for 1 h at room temperature, or with a human serum sample (diluted 1/300 in TBS) from a patient with clinically confirmed active TB, and incubated overnight at 4 °C. The bound antibodies were detected using HRP conjugated a goat anti-human IgG-Fc (Bethyl Laboratories, Inc., Montgomery, TX, USA, Cat. No. A80-104P-92) at 1/12,000 dilution in TBS for 2 h at room temperature. The purified rPstS-1 proteins from *P. pastoris* and *E. coli* were quantified using a Bradford protein assay (Bio-Rad, Richmond, CA, USA) following the supplier’s recommendations. Immunoreactive bands were detected by chemiluminescence (Thermo Scientific, SuperSignal^®^ West Pico Chemiluminescent Substrate), and band visualization was performed using a C-digital scanner (LI-COR, NE, USA). Densitometric analysis was performed using Image Lab™ Software 6.0.1 (Bio-Rad).

### Purification of rPstS-1

After 73 h of cultivation of the recombinant *P. pastoris* in the presence of methanol in the bioreactor, biomass clarification was carried out by tangential filtration (Sartojet pump, loaned by Sartorius Stedim, Gotinga, Germany), using a membrane with a 100 kDa cut off (Sartojet, Sartorius); phenylmethylsulfonyl fluoride (PMSF, Merck) was added to the filtrate to a final concentration of 0.1 mM. Following this, a membrane with a 10 kDa cut-off was used to concentrate the previous filtrate. The retained fraction (10–100 kDa) was concentrated by lyophilization of up to 90% of the initial volume, and then filtered using a 0.2 μm polypropylene filter (Minisart Sryinge filter, Sartorius). Finally, rPstS-1 was purified by reverse phase high performance liquid chromatography (RP-HPLC, Shimadzu, Kyoto, Japan). The samples were separated using a PROTO300 Semi-Prep C4 column (10 μm, 250 × 10 mm) and XBridge Protein BEH C4 column (300 Å, 3.5 mm, 4.6 mm × 150 mm). The composition of the solvents used to equilibrate the columns was as follows: buffer A contained Milli Q water with 0.1% trifluoroacetic acid and buffer B contained acetonitrile with 0.1% trifluoroacetic acid. The recombinant protein was eluted with a gradient of acetonitrile. The separation started with 20% of buffer B solution for 5 min, then a gradient of buffer B from 20% to 60% was applied for the next 20 min. Elution was monitored by measuring the absorbances at 280 and 220 nm. Purity was evaluated from the chromatogram obtained from each run by analyzing the representative area of each peak.

The purification of *E. coli* rPstS-1 was carried out by affinity chromatography of the His-tagged-rPstS-1 using an FPLC system (Econo System, Bio-Rad Richmond, CA, USA) using a Ni–NTA Agarose (Qiagen, Venlo, Netherlands). The rPstS-1 was eluted with 100 mM NaCl, 50 mM Tris–HCl, 8 M urea, 250 mM imidazole, pH 5.9. The rPstS-1 was then further purified by reverse phase high performance liquid chromatography (RP-HPLC, Shimadzu, Kyoto, Japan), using a PROTO300 Semi-Prep C4 column (10 μm, 250 × 10 mm) and the XBridge Protein BEH C4 column (300 Å, 3.5 mm, 4.6 mm X 150 mm). The recombinant protein was eluted with a gradient of 0% to 60% of acetonitrile, using buffer A (Milli Q water containing 0.1% trifluoroacetic acid) and buffer B (acetonitrile containing 0.1% trifluoroacetic acid). Elution was monitored by measuring absorbances at 280 and 220 nm.

### Circular dichroism spectroscopy

The secondary structure of both forms of rPstS-1 (from *P. pastoris* and *E. coli*) were determined by far-UV circular dichroism (CD) spectroscopy using a Jasco J-715 spectropolarimeter (Jasco Inc., MD. USA). The CD spectra were obtained from samples dissolved in 50 mM Tris, 100 mM NaCl, 12 mM CaCl_2_ at pH 8, with protein concentrations of 125 and 250 μg/mL. Spectra were recorded from 190 to 260 nm at a scan speed of 20 nm/min and a response time of 1 s, with each spectrum representing the sum of four accumulations. All spectra were acquired at 25 °C in a 1-mm path length cell. The CD data were analyzed using the CAPITO CD Analysis & Plotting Tool (http://capito.nmr.leibniz-fli.de/index.php) to provide estimates of the secondary structure content [93]. The mean residue ellipticity ([Θ] in grad.cm^2^.dmol^−1^) was calculated in accordance with the equation [107]: [Θ] = MRWΘ/10*d*.*c* where MRW is the mean residue weight, Θ is the observed ellipticity (millidegrees), *d* is the pathlength (cm), and *c* is the concentration (mg/mL).

### Mass spectrometry analysis

The molecular mass of *P. pastoris* rPstS-1 was determined by MALDI-TOF, using a Bruker Microflex instrument equipped with a 20-Hz nitrogen laser at I = 337 nm. Approximately 500 fmol of rPstS-1 and α-cyano-4-hydroxycinnamic acid (10 mg/mL) solutions were mixed (1:1 v/v) and spotted onto stainless steel plates. Samples were analyzed in the positive ion detection and linear mode; 70 laser shots were integrated into a single mass spectrum.

Sequence identification of the *P. pastoris* rPstS-1 purified by RP-HPLC was carried out as previously described [108]. Briefly, 50 μg of rPstS-1 was reduced with 10 mM DTT in 100 mM NH_4_HCO_3_ for 5 min at room temperature and then alkylated with 54 mM iodoacetamide in 100 mM NH_4_HCO_3_ at room temperate for 15 min in dark. After reduction and alkylation, the protein was digested with trypsin (recombinant proteomics grade, Roche, Cat. No. 03708985001) dissolved in 100 mM Tris, pH 8.5 and 1 mM CaCl_2,_ at a 1/50 ratio of enzyme to protein weight. The enzymatic reaction was performed for 18 h at 37 °C. On the other hand, a second treated sample (reduced and alkylated) containing 50 μg of purified protein was digested with the proteolytic enzyme Lys-C (Roche, Switzerland) in digestion buffer (25 mM Tris HCl, 1 mM EDTA, pH 8.5,) at a 1/100 ratio of enzyme to protein weight. The enzymatic reaction was performed for 16 h at 37 °C. Mass spectrometry analyses of the digested peptides were performed with 1 μL of the enzymatic reaction mixed with 5 μL of 30% acetonitrile (ACN), 70% water, 0.1% trifluoroacetic acid (TFA) saturated with α-cyano-4-hydroxycinnamic acid. Following this, 1 μL of this solution was analyzed in a matrix assisted laser desorption ionization time-of-flight analyzer (MALDI-TOF, Bruker Microflex) equipped with a 20-Hz nitrogen laser at I = 337 nm. Spectra were recorded in the linear positive mode for the mass range 1000 to 13,000 Da.

The identification of the sequence of tryptic peptides was performed using a MALDI-TOF-TOF 4800 (Applied Biosystems, Foster City, CA, USA). MS/MS experiments were carried out at 1 kV with collision-induced dissociation using air as the collision gas. Digested peptides were reconstituted in a solution of H_2_O:ACN:TFA (50:50:0.1) at nanomolar concentrations. In order to crystalize the peptides, 0.5 μL of the sample was mixed with 1.0 μL of the matrix α-cyano-4-hydroxycinnamic acid as also with with 1.0 μL of the matrices 3,5-dimethoxy-4-hydroxycinnamic acid and α-cyano-4-hydroxycinnamic acid. Both preparations were done in micromolar concentrations and deposited on stainless steel plates at room temperature until complete evaporation and simultaneous crystal formation. To ionize samples, laser pulses using a nitrogen source and a 355 nm wavelength were used. The duration, intensity, and number of pulses were adjusted depending on the matrix used and the ease of ionization of the sample, which is directly related to the chemical structure of the molecules. The detected signals were stored and analyzed with the ProteinPilot 1.4 program coupled to the system.

### Sero-reactivity experiments by ELISA

A 96-well microplate (Nunc-ImmunoPlate, Maxisorp Surface, Thermo Fisher) was coated with 100 μL/well of the recombinant protein (5 μg/mL) in bicarbonate buffer solution (PBS) and incubated overnight at 4 °C, then washed three times for 1 min each with PBS, blocked with 150 μL of BSA (0.25%) in PBS for 1.5 h at 37 °C, and then washed five times with PBS. Following this, 100  μL of each serum, diluted in blocking solution (at two dilutions; 1:100 and 1:500), was added to the appropriate well and incubated for 1.5 h at 37 °C and then washed three times with 250 μL of Tween 20 (0.05%) in PBS. The wells were then incubated with 100 μL of secondary antibody mouse anti-human IgG2 (1:500 PBS/Tween 0.05%) coupled to alkaline phosphatase (Thermo Fisher Scientific), for 1.5 h at 37 °C in the dark and washed four times with 250 μL of Tween 20 (0.05%) in PBS. To measure the alkaline phosphatase activity, SIGMAFAST™ p-nitrophenyl phosphate tablets were used as a substrate (Merck, Billerica, MA, USA). The diluted substrate (75 μL) was added to each well followed by incubation for 30 min in the dark at room temperature. After the incubation period, the absorbance was measured at 405 nm in a plate reader (Synergy|HTX, Biotek, Winooski, VT, USA). Each serum analysis was performed in duplicate. The sera from TB patients (n = 30) were randomly selected from the National Institute of Respiratory Diseases (INER, Mexico City, Mexico). Active pulmonary TB in all patients was confirmed by a clinically positive sputum smear, microscopy, culture in medium Middlebrook 7H10, and identification by molecular methods and resistance to rifampicin using the GeneXpert test. All sera were collected before starting the antibiotic treatment, after the confirmation of the acute phase of the disease. Additionally, the sera from subjects with positive (n = 5) and negative (n = 5) PPD tests were selected from a screening to evaluate mycobacterial infections in people from the State of Mexico, Mexico. These subjects did not present any clinical evidence of active tuberculosis.

### Statistical analyses

All the data for kinetic parameters are represented as the mean of triplicates ± standard deviation. Statistical significance between groups in the antibody reactivity assay was determined using the mean of 30 sera from patients with active TB compared with PBS as the control, and positive and negative PPD groups (n = 5, each). Dunn´s test was used for all pairwise comparisons and comparisons against a control group (PBS) following rank-based ANOVA, based in the treatment of unequal group sizes. Differences were considered statistically significant if the P < 0.05.

## Additional files

**Additional file 1: Figure S1.** Peptide sequencing by MS/MS. **A**) Shows a sequence corresponding to the peptide detected at m/z 1765.82 in the MALDI peptide mass map (Fig. 4C) revealed the sequence of P7T (Table 4). **B**) Presents a sequence corresponding to the peptide detected at m/z 4916.29 in the MALDI peptide mass map (Fig. 4C) revealed the sequence of P9T (Table 4). **C**) A sequence corresponding to the peptide detected at m/z 2272.13 and 2271.81 in the MALDI peptide mass map (Figure 4B, C) revealed the sequence of P13T (Table 4). **D**) Present the sequence corresponding to the peptide detected at m/z 1993.08 and 1994.49 in the MALDI peptide mass map (Fig. 4B, C) revealed the sequence of P14T (Table 4).

**Additional file 2: Figure S2.** Comparison of antibody recognition of *P. pastoris* rPstS-1 and *E. coli* expressed His-tag-rPstS-1. Western blotting performed of rPstS-1 produced in *P. pastoris* (lanes 1, 2 and 3, using 2.0, 1.0 and 0.5 μg of rPstS-1, respectively) or *E. coli* (lanes 4, 5 and 6, using 2.0, 1.0 and 0.5 μg of rPstS-1, respectively), incubated with **A**) a rabbit anti-*Mycobacterium tuberculosis*-PstS-1 polyclonal antibody and **B**) a human serum sample from patient clinically confirmed active TB. For each blot, molecular mass markers (kDa) are indicated on the right.
