# Supplementary figures and images for: Recombinant O-mannosylated protein production (PstS-1) from Mycobacterium tuberculosis in Pichia pastoris (Komagataella phaffii) as a tool to study tuberculosis infection
Source: Microb Cell Fact. 2019 Jan 19;18:11. doi: 10.1186/s12934-019-1059-3 (PMC6339365; doi:10.1186/s12934-019-1059-3)

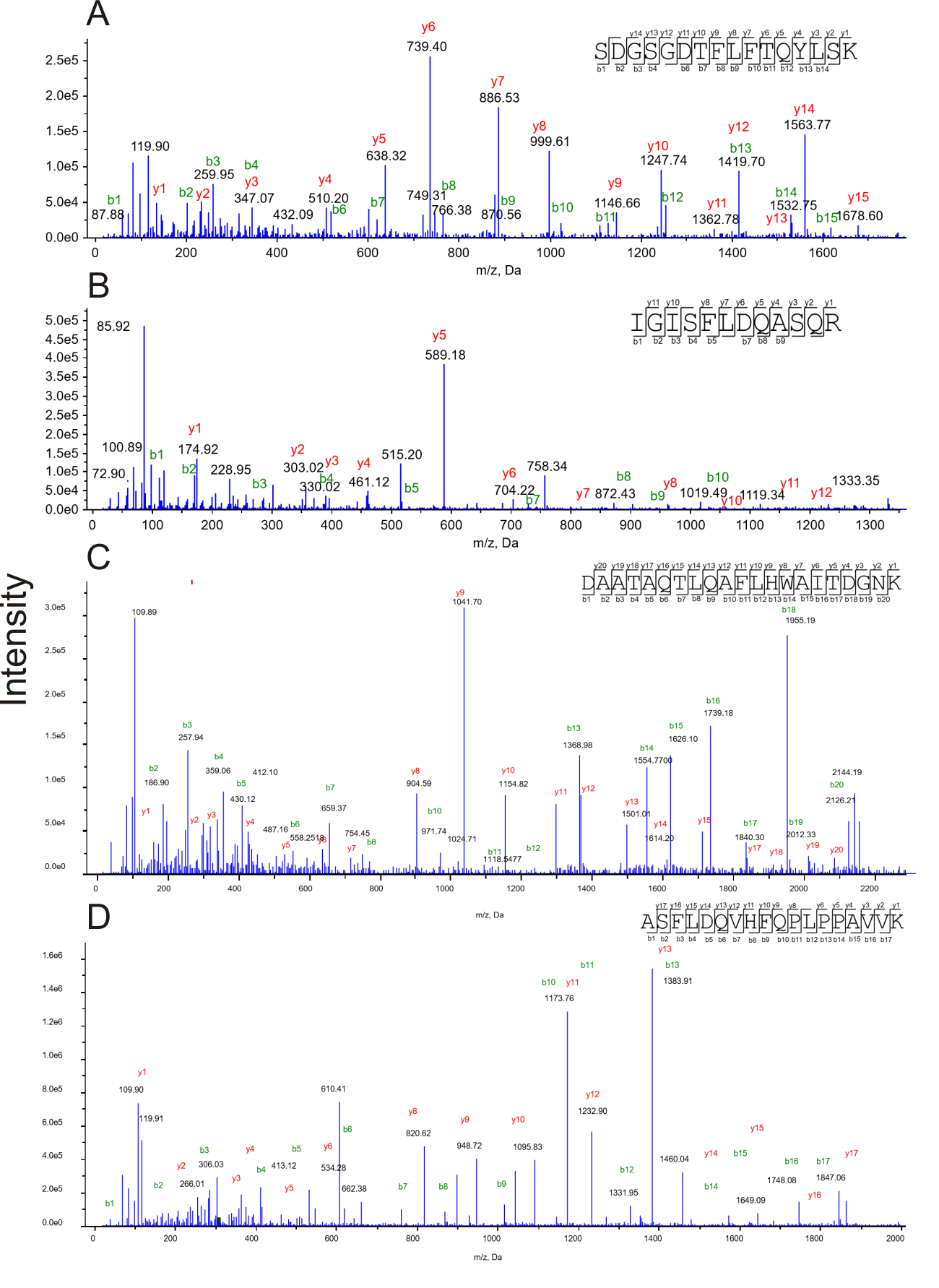

Supplement: Supplementary file 1 — Additional file 1: Figure S1. Peptide sequencing by MS/MS. A) Shows a sequence corresponding to the peptide detected at m/z 1765.82 in the MALDI peptide mass map (Fig. 4C) revealed the sequence of P7T (Table 4). B) Presents a sequence corresponding to the peptide detected at m/z 4916.29 in the MALDI peptide mass map (Fig. 4C) revealed the sequence of P9T (Table 4). C) A sequence corresponding to the peptide detected at m/z 2272.13 and 2271.81 in the MALDI peptide mass map (Figure 4B, C) revealed the sequence of P13T (Table 4). D) Present the sequence corresponding to the peptide detected at m/z 1993.08 and 1994.49 in the MALDI peptide mass map (Fig. 4B, C) revealed the sequence of P14T (Table 4). [file 12934_2019_1059_MOESM1_ESM.tif]

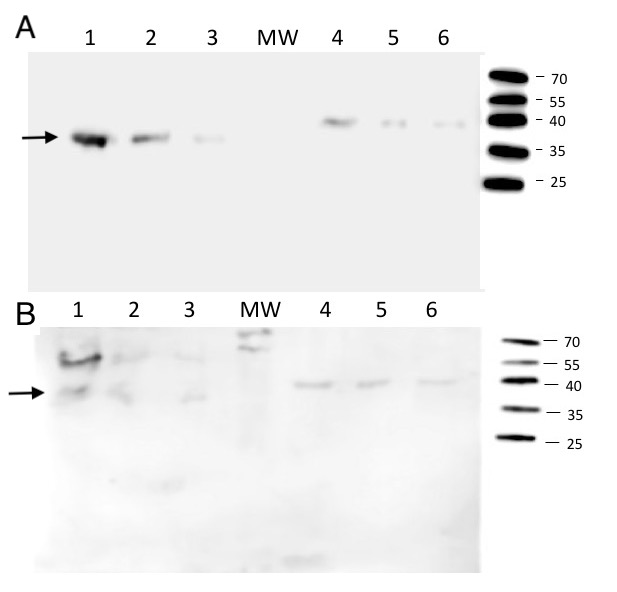

Supplement: Supplementary file 2 — Additional file 2: Figure S2. Comparison of antibody recognition of P. pastoris rPstS-1 and E. coli expressed His-tag-rPstS-1. Western blotting performed of rPstS-1 produced in P. pastoris (lanes 1, 2 and 3, using 2.0, 1.0 and 0.5 μg of rPstS-1, respectively) or E. coli (lanes 4, 5 and 6, using 2.0, 1.0 and 0.5 μg of rPstS-1, respectively), incubated with A) a rabbit anti-Mycobacterium tuberculosis-PstS-1 polyclonal antibody and B) a human serum sample from patient clinically confirmed active TB. For each blot, molecular mass markers (kDa) are indicated on the right. [file 12934_2019_1059_MOESM2_ESM.jpg]
